# Oligometastatic non-small cell lung cancer: Current management

**Published:** 2021-05-27

**Authors:** Alicia Román-Jobacho, María Hernández-Miguel, María Jesús García-Anaya, Jaime Gómez-Millán, J. A. Medina-Carmona, Ana Otero-Romero

**Affiliations:** ^1^Department of Radiation Oncology, Hospital Virgen de la Victoria, Málaga, Spain; ^2^Hospital Rey Juan Carlos, Móstoles, Madrid

**Keywords:** lung cancer, oligometastases, stereotactic

## Abstract

**Background:**

In the past decade, major developments have improved the survival of patients with oligometastatic non-small cell lung cancer (NSCLC). About 20% - 50% of patients with NSCLC present with oligometastases at diagnosis. For this group of patients, it seems that an increase in survival would justify aggressive local therapies. The development of minimally invasive surgery and advanced radiotherapy techniques like stereotactic body radiation therapy (SBRT) makes local control possible for selected patients with metastatic NSCLC. The advantage of SBRT over surgery is that it is a non-invasive technique, with minimum side effects, and is more suitable for fragile and elderly patients, non-candidates for surgery, or patients who refuse surgery.

**Aim:**

The purpose of this review is to summarize the latest scientific evidence on the management of oligometastatic NSCLC, focusing on the role of radiotherapy.

**Relevance for Patients:**

The initial treatment recommended for patients with oligometastatic NSCLC is systemic therapy. Patients should be considered for radical treatment to both the primary tumor and oligometastases. Aggressive local therapy comprises surgery and/or definitive radiotherapy such as SRS or SBRT, and may be preceded or followed by systemic treatment. Recent clinical evidence from Phase II trials reports benefits in terms of PFS in patients with good performance status and long disease-free periods, with good response to systemic therapy, especially in EGFR wild-type tumors. Phase I and II trials have shown that radiotherapy combined with immunotherapy can improve tumor response rate and possibly overall survival. The recommendation is also to include OM patients in ongoing clinical trials.

## 1. Introduction

Stage IV non-small cell lung cancer (NSCLC) has traditionally been considered an incurable disease, with low life expectancy, even when treated with standard chemotherapy based on platinum. However, in the past decade, major advances have improved the survival of these patients, especially those with oligometastatic NSCLC (OM-NSCLC)

In 1995, Helmann and Weichselbaum were the first to describe the oligometastatic state as an intermediate state between localized disease and widespread disease, with a more indolent biology and lower dissemination capacity [1]. The 8^th^ edition of American Joint Committee on Cancer reclassified Stage IV NSCLC based on the number of metastases, with M1b an extrathoracic metastasis (IVA) and M1c multiple extrathoracic metastases in one or more organs (IVB) [2].

This new classification divides groups of patients according to different therapeutic approaches and prognoses. In NSCLC, the oligometastatic state is relatively common, with 20–50% of patients having oligometastatic disease at diagnosis.

It seems that for this group of patients, an increase in survival would justify aggressive local therapies [3].

Initially, the only radical treatment for oligometastases was surgery. The development of minimally invasive surgery and advanced radiotherapy techniques like stereotactic body radiation therapy (SBRT) makes local control (LC) possible for selected patients with metastatic NSCLC. SBRT allows the treatment of lesions in any part of the body, with a small number of fractions and a minimum toxicity profile. It also has an advantage over surgery in that it is a non-invasive technique, with minimum side effects, and is more suitable for fragile and elderly patients, non-candidates for surgery, or patients who refuse surgery.

In an individual patient meta-analysis of 757 patients with NSCLC, Ashworth *et al*. found that most oligometastases were in the brain (35.5%) or lung (33.6%), followed by the adrenal gland (13.0%), bone (8.5%), other (7.8%), liver (2.4%), and lymph node (2.4%) [4]. The meta-analysis revealed that the median overall survival (OS) in patients treated with ablation of all disease sites including the primary tumor was 26 months, and survival at 1, 2, 5, and 8 years was 70.2%, 51.1%, 29.4%, and 23.4%, respectively [5].

The purpose of this review is to summarize the latest scientific evidence on the management of oligometastatic NSCLC, focusing on the role of radiotherapy.

## 2. Definitions of Oligometastases

There is no well-established number of metastases to define a patient as oligometastatic. A European multidisciplinary consensus statement on the definition and staging of OM-NSCLC considered that the maximum number of metastases/organs involved depends on the possibility of offering a radical-intent treatment strategy. Based on the systematic review, a maximum of five metastases and three organs was agreed on. The presence of diffuse serosal metastases or bone marrow involvement excludes cases from this definition [6].

There are additional suggested definitions that cover different scenarios in the presentation of oligometastases.

- “***De novo* oligometastases”** or **“synchronous oligometastasis**” refers to the presence of a limited number of metastases at diagnosis. Patients with widely disseminated disease at diagnosis who present with oligometastases after systemic treatment are said to have **“induced oligometastases”** [1]
- The term oligorecurrence refers to progression with new metachronous oligometastases after definitive treatment of the primary locoregional thoracic disease [7]
- Oligopersistent disease is a concept that refers to oligometastatic patients who remain stable after systemic therapy or who, starting from a more widespread disease, achieve an oligometastatic state [8]
- Oligoprogressive disease describes a situation in which patients with disseminated disease at diagnosis respond to systemic treatment, remaining stable while one or a limited number of metastases progress during systemic therapy [9].

The frequency of oligoprogression during treatment with tyrosine kinase inhibitors (TKIs) varies according to the definition used, however, estimates vary from 15% to 47% [10,11]. It is believed that oligoprogression arises as a result of tumor heterogeneity and the development of isolated resistance in one or more metastatic sites [12].

The prognosis among these clinical situations is different, and each represents a heterogeneous group contingent on the number of metastatic lesions, tumor genotype (EGFR mutated, ALK rearrangements, and so forth), and type of systemic treatment [13].

## 3. Local Ablative and/or Systemic Therapy to Synchronic Oligometastatic State

Two randomized Phase II trials have shown an increased PFS by adding radical local treatment to systemic therapy in patients with oligometastatic NSCLC who achieve good response [14,15].

Nevertheless, many questions remain regarding the treatment of these patients, which patients receive the greatest benefit from ablative treatment.

If not previously performed, Stage IV NSCLC patients should undergo evaluation with brain MRI or CT scan and whole-body PET scan to ensure oligometastatic status. Although most studies were conducted before the PET scan era, recommendations are to perform such an evaluation because approximately 15% of patients with NSCLC initially classified as Stage I-III by CT scan will change classification to Stage IV with a PET scan [16].

National Comprehensive Cancer Network guidelines include the recommendation of a confirmatory biopsy of the metastatic lesion whenever possible [4,17].

In a recent multi-institutional Phase II study, Gomez *et al*. observed an improvement in OS after aggressive local treatment of NSCLC patients with three or fewer metastatic lesions that had not progressed to the first line of chemotherapy. The trial closed early due to the statistically significant median progression-free survival advantage for patients in the local consolidation arm of 14.2 months versus 4.4 months. Later, long-term results showed a significantly longer median OS for these patients (41.4 months vs. 17 months) [14].

A subsequent randomized Phase II trial showed similar favorable results in 29 patients with five or fewer metastases and partial response or stability after systemic treatment. All lesions, including primary lesions, were treated with SBRT or hypofractionated radiation therapy followed by maintenance chemotherapy versus maintenance alone [15]. This trial also closed early due to better disease-free survival (DFS) in the consolidated local therapy arm, with a median DFS of 9.7 months compared to 3.5 months. In addition, no patient with local treatment developed progression in an irradiated site, as opposed to 70% in the exclusive maintenance group.

Surgery is an established treatment for cerebral, pulmonary, and adrenal oligometastases of lung cancer.

Occasionally, patients with isolated metastases in other locations (e.g. bone and liver) have received treatment with metastasectomy; however, the number reported is much smaller as this treatment may not be suitable for patients with several affected locations. Altogether, systemic therapies alone may not be optimal in disease settings such as oligometastatic lung cancer, where long-term control can be expected. Kissel *et al*. assessed the outcomes of 91 lung cancer patients with extracranial metastases in oligometastatic, oligorecurrent, oligopersistent, and oligoprogressive settings (the “oligometastatic spectrum”) under strategies using SBRT ± systemic treatments. With a median follow-up of 15.3 months, crude LC at irradiated metastases was 91%, whereas median distant progression-free survival (dPFS) oligoprogressive patients had the worst dPFS, and OS was 6.3 and 28.4 months, respectively (2-year survival 54%). Initial nodal stage and the oligometastatic spectrum were prognostic factors for dPFS; the multivariate analysis showed age, initial primary stage, and oligometastatic spectrum as prognostic factors for survival. For oligorecurrent patients, a longer free interval between primary and metastatic spread was associated with better survival, with a threshold of 2 years (*P*=0.002) [18].

Options for local therapy include surgery, SBRT, and radiofrequency. Due to the lack of randomized trials that compare these modalities, the choice of local therapy in oligometastases depends on several factors and a multidisciplinary team should take this decision based on:

- Patient-related factors including performance status, age, comorbidities, and patient decision
- Metastases-related factors such as size, number of lesions, resectability, and remaining volume of site organ
- Treatment-related factors: Technique availability, professional training, and costs.

There are several ongoing Phase II/III trials to assess the role of SBRT treatment in oligometastatic NSCLC combined with chemotherapy and/or immunotherapy. Results will be available in the near future, Table 1 [19].

**Table 1:** Selected ongoing clinical trials in metastatic NSCLC exploring radiotherapy to the primary tumor and metastatic disease

Clinical trial number	Study name	Patient characteristics
NCT03137771	Maintenance systemic therapy versus LCT Plus maintenance systemic therapy for limited metastatic non-small cell lung cancer (NSCLC): A Randomized Phase II/III Trial	NSCLC synchronous or metachronous oligometastatic (≤3 extracranial metastases)
NCT02417662	Stereotactic ablative radiotherapy for oligometastatic non-small cell lung cancer. A Randomized Phase III Trial (SARON)	NSCLC synchronous oligometastatic (≤3 metastases) EGFR/ALK negative or unknown mutational status
NCT03275597	Comprehensive SBRT to all sites of oligometastatic NSCLC combined with durvalumab (MEDI4736) and tremelimumab dual immune checkpoint inhibition	NSCLC synchronous oligometastatic ≤6 extracranial sites. EGFR/ALK negative
NCT03391869	Randomized Phase III trial of LCT after nivolumab and ipilimumab for immunotherapy-naive patients with metastatic non-small cell lung cancer (LONESTAR)	NSCLC poly- and oligometastatic EGFR/ALK-negative adenocarcinoma
NCT02893332	Tyrosine-kinase inhibitor with or without SBRT in newly diagnosed advanced staged lung adenocarcinoma	NSCLC synchronous or metachronous oligometastatic (≤5 metastases) with EGFR mutation

SBRT: Stereotactic body radiotherapy; LCT: Local consolidative therapy; NSCLC: Nonsmall cell lung cancer

No research reports direct comparisons between surgery and radiotherapy as a radical treatment for oligometastatic disease, but several studies have included patients treated with either surgery or radiotherapy. Evidence for the other ablative modality, radiofrequency ablation, is still limited. The advantages of radiofrequency ablation are the freedom to perform the procedure regardless of any previous therapy and its repeatability. However, rare but serious complications may occur, including bronchopleural fistula, pulmonary artery pseudoaneurysm, systemic air embolism, injury of the brachial nerve and phrenic nerve, pneumonia, and needle tract seeding of the cancer [13].

The ongoing Phase II STOP trial includes patients with NSCLC in oligoprogression and randomizes them to receive their standard systemic treatment or SBRT to all oligoprogression sites (maximum five). The primary endpoint is progression-free survival, and the expected completion date of this study is in 2021 [20].

## 4. Treatment of the Primary Tumor

Treatment of the primary tumor is a topic of debate. Justification for a radical approach to the primary tumor could be that distant spread of the tumor comes from both the primary tumor and metastases, so radical treatment to both could improve OS in OM NSCLC patients [21].

It is known that patients with a solitary site of metastasis, frequently the brain or adrenal gland, who undergo surgical resection of both the primary and the metastasis can occasionally experience long-term survival or even cure [22].

Petrelli *et al*. conducted a systematic review and meta-analysis to study the role of local radiation therapy to the primary tumor in synchronous OM NSCLC [23]. Their review included 21 studies: Four Phase II studies, two randomized Phase II studies, and 15 retrospective series. Radiotherapy was delivered with palliative doses in 1.4% of patients, the rest of cases received a treatment of 3D external radiotherapy or SBRT, either alone (21%), in combination with sequential or concomitant chemotherapy (79%), or adjuvant to surgery (0.4%). The median pooled OS was 20.4 months (95% CI 16.6–24.7) and median PFS was 12 months (95% CI 9.8–14.5). Thoracic radiation therapy improved OS (HR=0.44, 95% CI 0.32–0.6; *P*<0.001) and PFS (HR=0.42, 95% CI 0.33–0.55; *P*<0,001).

Li *et al*. conducted a meta-analysis to identify prognostic factors of OM NSCLC and found that the factors associated with better outcomes using a radical approach to the primary tumor were sex (female), (y)pT stage, negative nodal status, and adenocarcinoma histology [24].

The Phase II trial by Gomez *et al*. mentioned above compared outcomes in 49 OM NSCLC patients (≤3 metastases) with no progression after a minimum 4 cycles of platinum-based chemotherapy or 3 months of anti-EGFR/anti_ALK therapy. Patients were randomized to receive local radiotherapy followed by maintenance systemic therapy or observation versus only maintenance or observation. The trial found a significant benefit in median progression-free survival (PFS) in the consolidative local treatment arm (14.2 months vs. 4.4 months). Long-term results published in 2019 reported significantly longer median OS in consolidative local treatment (41.2 months vs. 17.0 months). Regarding local treatment, 67% of patients received radiation therapy, either concomitant with chemotherapy, exclusively hypofractionated radiotherapy or SBRT, while 33% of patients had surgery to the primary tumor. Radiotherapy regimens varied considerably, from palliative doses to ablative doses, but even lower doses of radiation provided a benefit in delaying progression [14].

Iyengar *et al*. explored the role of radiation therapy to both the primary tumor and the metastases in 29 patients with OM NSCLC, excluding EGFR- or ALK-mutated tumors. Patients with stable disease or partial response after 4–6 cycles of chemotherapy were randomized to receive SBRT to the metastases and radiation therapy (SBRT or hypofractionated RT) followed by maintenance chemotherapy versus maintenance chemotherapy alone. The trial closed early due to significantly better PFS in the consolidative local treatment arm (9.7 months vs. 3.5 months) [15].

## 5. Combination of Systemic Therapy and Radiotherapy

The current level of evidence does not support the routine use of local ablative treatment as the initial treatment in oligometastatic disease, for which systemic therapy remains the standard of care. Local treatment approaches could be considered for patients not suitable for systemic therapy or for those who refuse or want to delay it [8].

The most suitable treatment sequence remains a matter of controversy because data come from retrospective studies or small prospective studies.

Various factors require consideration, such as the time of diagnosis (synchronous vs. metachronous), primary tumor local extension, the number, size and location of the metastases, performance status, the absence or presence of symptoms, patient comorbidities, and the absence or presence of target mutations.

One accepted strategy in patients with synchronous metastases is to begin with systemic treatment and later evaluate disease response before radical treatment to the primary tumor as well as metastases.

After morphological diagnosis, the next consideration is therapy-predictive biomarker testing. It is well known that EGFR-mutated and ALK-translocated NSCLC treated with oncogene-driven targeted therapy has significantly better outcomes compared with standard platinum-based systemic therapy [25,26]. Acquired resistance to first-line TKI can occur on average after 9–12 months of treatment. Patterns of progression include generalized progression, brain progression, or oligoprogression. Oligoprogression is estimated to occur during TKI treatment in 15–47% of patients due to tumor heterogeneity and an isolated resistant subclone at 1–4 metastatic sites [12]. Data from retrospective studies suggest that aggressive local treatment can eradicate TKI-resistant oligometastases offering a benefit in LC. In this setting, the preference is for SBRT rather than surgery or normofractionated radiotherapy due to its shorter duration and minimal side effects. In this manner, SBRT allows continued use of TKI to control the majority of the disease [27].

In 2018, De Ruysscher *et al*. [28] conducted a single-arm prospective Phase II trial with 39 NSCLC Stage IV patients who had fewer than five metastases at primary diagnosis and management with radical local treatment (radiotherapy or surgery). No previous response to systemic treatment was required. Thirty-seven patients (95%) received chemotherapy. The median OS was 13.5 months and median PFS was 12.1 months. Three patients (7%) had a local relapse.

For patients with metastatic disease limited to the central nervous system, initiation of treatment with stereotactic radiosurgery (SRS) or surgical resection is an option; however, for patients with ALK or EGFR mutation who present asymptomatic lesions, it is reasonable to start with targeted therapy before radiotherapy treatment [29,30].

In the metachronous setting, the Phase II SABR-COMET demonstrated a survival benefit of SBRT as a consolidation treatment in the context of oligometastatic disease. The trial recruited patients with metachronous oligometastasis disease of any histology who had previously received treatment to the primary tumor. Patients treated with SBRT to metastases sites had better OS compared to systemic treatment alone [31].

The randomized prospective Phase II/III trial, NRG-LU002, examines maintenance systemic therapy alone compared with SBRT to all metastatic sites (three or more), plus radiation to the primary site followed by systemic therapy to determine the benefit of treatment of all metastases in NSCLC. Primary outcomes include PFS and OS. The UK conventional care versus radioablation (stereotactic body radiotherapy) for extracranial oligometastases trial is a multicenter, randomized, Phase II trial investigating whether the addition of SBRT to standard therapy in patients with oligometastatic NSCLC, breast, renal cell, or prostate cancer (three or more extracranial metastases) will improve PFS, OS, LC, and freedom from widespread metastatic disease. It will also collect toxicity and quality-of-life data. These data will represent some of the first available robust randomized evidence regarding the use of metastasis-directed local therapy in the setting of oligometastatic NSCLC [32,33].

## 6. Immunotherapy and Ablative Radiotherapy

Patients with oligometastatic lung disease are commonly treated with immunotherapy, and findings show that ionizing radiation can produce greater antigen presentation to better immune system recognition [34].

Unfortunately, not all patients respond to this treatment, possibly because tumor antigens are not recognized.

Ablative doses of SBRT can induce necrosis and senescence, types of cell death in which there is a greater release of tumor antigens and higher infiltration of T lymphocytes in the irradiated tumor with the release of cytokines related to cell damage [35-37].

SBRT may synergize with immunotherapy; the combination of both treatments shows the production of more tumor regression in several solid tumor types.

Ionizing radiation can also increase the effect of immune checkpoint inhibitors such as pembrolizumab or durvalumab. Thus, some authors have postulated that SBRT before immunotherapy can lead to the improved efficacy of this immunological treatment [38].

Phase I and II trials have tested the safety of the SBRT and immunotherapy combination. In 2014, Tang published results of the first Phase I/Phase II trial that combined SBRT with immunotherapy in patients with advanced NSCLC. The study observed stable disease or partial response in 67% of patients, and patients who received sequential radiation to lung metastases had better OS than those who received sequential radiation to liver metastases [39,40].

In 2015, the KEYNOTE-001 trial demonstrated the efficacy and safety of durvalumab in advanced lung cancer. A secondary analysis found that patients who received radiotherapy prior to pembrolizumab treatment doubled OS (10.7 vs. 5.3 months) [41,42].

The PEMBRO-RT Phase II trial published its results in July 2019. The trial included patients with recurrent metastases after at least one regimen of chemotherapy. Patients were randomized to either pembrolizumab alone, a selective humanized PD-1 monoclonal antibody, or pembrolizumab after SBRT on a single tumor site. Patients had at least two separate metastatic lesions, only one of which was irradiated, and response was measured in both locations to test the immunological effect in the non-irradiated area. Although the overall response rate at 12 weeks was 18% in the control arm versus 36% in the experimental arm, these results did not meet the study criteria for meaningful clinical benefit. A subgroup analysis revealed that the patients who benefited the most, in terms of progression-free survival and OS, were those with tumors that did not express PD-L1, suggesting that combined treatment improved antigen presentation and response [43].

A subsequently published pooled study that analyzed the results of PEMBRO-RT together with the MDACC study [44], which had similar inclusion criteria and treatment schemes, showed the best out-of-field response rate with radiotherapy plus pembrolizumab versus pembrolizumab alone (41.7% vs. 19.7%, *P*: 0.0039). However, longer median progression-free survival (9 months vs. 4.4 months, *P*: 0.04) and significantly better median OS (19.2 months vs. 8.7 months, *P*: 0.0004) occurred among the radiotherapy group [45].

Hence, it would appear that adding radiotherapy to immunotherapy can increase responses and outcomes in patients with metastatic lung cancer, although validation in a randomized Phase 3 trial is necessary.

## 7. Treatment of Oligometastases by Site

### 7. 1. Brain oligometastasis

Approximately 25–30% of patients who have lung cancer present with brain metastases at diagnosis. In certain situations, such as a limited number of metastases, favorable histology, and good performance status, the radical management of brain metastases is feasible, which translates into better outcomes [46,47].

Brain-only oligometastases represent a subgroup with better prognosis if radical treatment to primary and cerebral lesions is possible, even in node-positive disease, and prognosis is similar to Stage III with definitive chemoradiotherapy, with a median OS <24 months and 3-year OS of 40% [48].

The most important treatment approaches in this situation are surgery, whole-brain radiotherapy (WBRT), and SRS. Clinicians do not use WBRT as often in patients with limited brain metastases due to concerns about long-term toxicity, which includes neurocognitive effects.

### 7. 2. Adrenal gland oligometastasis

Adrenalectomy was the first treatment modality used in the management of adrenal metastases, although SBRT is beginning to offer a safe and effective alternative in non-operable cases.

Current evidence comes from retrospective studies, and the number of fractions and doses prescribed varies among the series (10–45 Gy in 3–10 fractions) [13].

Chen *et al*. issued an analysis of 39 studies published between 2009 and 2019, reporting outcomes of 1006 patients. The pooled 1- and 2-year rates of LC were 82% (74–88%) and 63% (50–74%), respectively, and the pooled 1- and 2-year OS rates were 66% (57–74%) and 42% (31-53%), respectively. There was a strong positive association between SBRT dose and 1- and 2-year LC (*P*<0.0001 and *P*=0.0002), and an association with 2-year OS (*P*=0.03). The overall rate of Grade 3 or higher toxicity was 1.8%. They concluded that SBRT for adrenal metastases was a safe treatment associated with excellent 1-year LC, the effective palliation of pain and a reduction of tumor volume. The ideal dose is still under investigation [49].

### 7. 3. Liver oligometastases

At present, surgery is the local treatment of choice for liver metastases and it achieves a LC of 85–90%; however, only 10-20% of patients with liver metastases are candidates for surgery [13]. There are few reported cases of liver metastasis resection from lung cancer, but long-term survival is high (21–60 months), which can be explained by patient selection [50,51].

Despite surgery being contraindicated for most liver metastases from lung cancer, radiotherapy can be a local ablative treatment option when indicated.

Many prospective and retrospective studies [52] have analyzed SBRT in liver oligometastases. All of the studies included patients with liver metastases from different primary tumors. LC at 2 years was around 90% and had a low toxicity profile, doses ranged from 36 to 60 Gy in 3 to 6 sessions. Rusthoven’s Phase II study showed an OS of 12 months for the unfavorable group [53-55].

With the scientific evidence currently available, high LC rates with SBRT would seem to be similar to those obtained using other local treatments, however, the results of the prospective studies currently in progress may alter these findings.

Thus, patients with 1–3 hepatic lesions can benefit from aggressive local treatment with SBRT to improve LC and possibly improve survival.

### 7. 4. Lung oligometastases

The metastasectomy study based on 5206 cases of different histologies published its results in 1997 [56]. With a mean follow-up of 46 months, actuarial survival at 5, 10, and 15 years was 36%, 26%, and 22%, respectively.

However, only a few patients were candidates for surgery. After lobectomy, LC rates ranged from 85% to 95%, and after wedge resections, LC rates ranged from 50% to 70% [57,58].

Phase I and II trials of SBRT for primary tumors and lung oligometastases have demonstrated feasibility, safety, and efficacy with good to excellent LC in most studies. LC rates reported using SBRT are in the range of 70–90% at 2 years, which are similar to those obtained with metastasectomy [59].

Rieber *et al*. reviewed 700 patients with medically inoperable lung metastases secondary to different malignancies treated with SBRT in 20 German centers between 1997 and 2014. They evaluated primary and metastatic tumor characteristics, treatment characteristics, and follow-up data including survival, LC, distant metastases, and toxicity. Lung metastases were treated with median PTV-encompassing single doses of 12.5 Gy (range 3.0–33.0 Gy) in a median number of three fractions (range 1–13). Two-year LC and OS were 81.2% and 54.4%, respectively. Independent prognostic factors for LC were performance status and biological effective dose at both isocenter and periphery. Survival was significantly better for patients with a good performance status, small and single pulmonary metastases, a long time interval between primary tumor diagnosis and SBRT treatment, and a favorable primary tumor histology [60].

Ashworth *et al*. carried out a meta-analysis of 757 patients with NSCLC with 1–5 synchronous or metachronous metastases treated with surgical metastasectomy, stereotactic radiotherapy/radiosurgery, or radical external beam radiotherapy, and curative treatment of the primary lung cancer, from hospitals worldwide. They observed significant OS differences in oligometastatic patients depending on the time of the oligometastatic presentation; thus, survival was better in metachronous disease than in synchronous disease (*P*<0.001) [5].

At present, there are no randomized trials that compare SBRT and surgery in the treatment of lung metastases, however, both treatments seem to have similar LC.

### 7. 5. Other locations

SBRT for other locations of oligometastases, such as bone, spinal cord, and lymph nodes, has been gaining widespread acceptance given the low toxicity profile and excellent LC rates reported in retrospective studies, which are as high as 85-100%. Doses administered with SBRT vary among the different series, making comparisons difficult. Prospective studies are necessary to clarify the most appropriate dose and timing of SBRT in these locations [61,62].

## 8. Conclusion

In general, the recommendation is for systemic therapy as the initial treatment for patients with oligometastatic NSCLC. Aggressive local therapy comprises surgery and/or definitive radiotherapy such as SRS and SBRT, and may be preceded or followed by systemic treatment. Patients should be considered for radical treatment to both the primary tumor and oligometastases. The selection of patients who might clearly benefit from a radical approach is challenging, and a multidisciplinary team should discuss such a decision. Recent clinical evidence from Phase II trials reports benefits in terms of PFS in patients, with good performance status and long disease-free periods, and a good response to systemic therapy, especially in EGFR wild-type tumors.

Phase I and II trials have shown that radiotherapy combined with immunotherapy can improve tumor response rate and possibly OS. The recommendation is also to include OM patients into ongoing clinical trials.

### Conflicts of interest

All the authors declare that there are no conflicts of interest.
